# Decoding Pigeon Behavior Outcomes Using Functional Connections among Local Field Potentials

**DOI:** 10.1155/2018/3505371

**Published:** 2018-02-15

**Authors:** Yan Chen, Xinyu Liu, Shan Li, Hong Wan

**Affiliations:** ^1^School of Electrical Engineering, Zhengzhou University, Zhengzhou, China; ^2^School of Information Engineering, Huanghuai University, Zhumadian, Henan, China; ^3^Henan Key Laboratory of Brain Science and Brain-Computer Interface Technology, School of Electrical Engineering, Zhengzhou University, Zhengzhou, Henan, China

## Abstract

Recent studies indicate that the local field potential (LFP) carries information about an animal's behavior, but issues regarding whether there are any relationships between the LFP functional networks and behavior tasks as well as whether it is possible to employ LFP network features to decode the behavioral outcome in a single trial remain unresolved. In this study, we developed a network-based method to decode the behavioral outcomes in pigeons by using the functional connectivity strength values among LFPs recorded from the nidopallium caudolaterale (NCL). In our method, the functional connectivity strengths were first computed based on the synchronization likelihood. Second, the strength values were unwrapped into row vectors and their dimensions were then reduced by principal component analysis. Finally, the behavioral outcomes in single trials were decoded using leave-one-out combined with the *k*-nearest neighbor method. The results showed that the LFP functional network based on the gamma-band was related to the goal-directed behavior of pigeons. Moreover, the accuracy of the network features (74 ± 8%) was significantly higher than that of the power features (61 ± 12%). The proposed method provides a powerful tool for decoding animal behavior outcomes using a neural functional network.

## 1. Introduction

The local field potential (LFP) is a low-pass filtered signal and it is considered to primarily reflect the sum of the slow local synaptic currents originating from an area around the electrode tip [1]. In recent years, the LFP has received increasing attention for several reasons. First, the LFP has been shown to reflect sensory and motor-related signals that can be modulated by cognitive processes, and it provides additional information regarding single neuron activity [2]. Second, the LFP appears to be correlated more closely with the BOLD signal measured by fMRI than spike activity [3–5]. Third, the LFP has been shown to correspond to spike activity under certain behavioral or perceptual conditions [6]. Finally, the LFP is easy to record, even over long periods of time, so it may be an efficient candidate signal for the control of neural prostheses [7].

Recently, an increasing number of studies have employed the LFP to decode different behavioral outcomes and to control the hand's trajectory via a brain-machine interface. For example, Scherberger et al. [2] showed that the LFP can predict an animal's intended movements based on single-trial information. Hwang and Andersen [7] also showed that single-channel LFP provided more accurate target information than single-channel spikes, and decoders using LFPs outperformed decoders using only spikes. In addition, the LFP has been used in research and disease diagnostics for decades, such as Parkinsonian tremor identification [8] and the diagnosis of Alzheimer's disease [9]. However, surprisingly little is known about the functional connectivity properties among LFPs and their relationships with behavioral outcomes.

In the last few years, functional connectivity has become a useful tool for exploring information related to directed functional interactions and the mechanism responsible for their integration in neuroscience. In practical applications, neural functional networks can be represented as a graph comprising statistical correlations between neural signals, where the nodes may be neurons or cortical areas, and the networks can be weighted or unweighted, directed or undirected. Xie et al. [10] demonstrated that the functional connectivity strengthened and the information transfer efficiency increased during a rat working memory task. Lu et al. [11] described a network-based method for predicting the behavioral outcomes of single trials in rat memory tasks. In addition, many network features have also been introduced as neurophysiological biomarkers of disease in clinical application. For example, the small-worldness of functional networks can be used as a diagnostic criterion for Alzheimer's disease [12–14]. Thus, neural functional network analysis may be a useful tool for decoding behavior in animals.

Evidence suggests that the prefrontal cortex (PFC) is critical for goal-directed behavior in mammals [15–17], where it organizes goal-directed actions. The avian nidopallium caudolaterale (NCL) resembles the PFC in terms of its anatomical connectivity [18], neurochemical organization [19], receptor architecture [20], and functional characteristics [21–23]. Studies have shown that [11, 24] neural activities can represent behavioral information in the mammalian PFC with different firing patterns. Several studies [10, 11, 25] have employed the features of spike functional networks to decode different behavioral outcomes. However, it is not clear whether the functional connectivity of LFPs from the avian NCL can be used to represent behavioral information. In addition, it is not known whether LFP network features can decode the behavioral outcome in a single trial.

To address these problems, we developed a network-based feature extraction method to decode pigeon behavior outcomes in single trials during a goal-directed decision-making task. In the task, pigeons were trained to turn left, move forward, or turn right at the choice point in a maze with food at the ends of the reward arms. The LFP signals were recorded from the NCL during turning by pigeons. The LFP spectral properties were then analyzed and the feature band was extracted using a wavelet filter. A neural functional network was constructed using a synchronization likelihood (SL) index [26]. The strength values of the network connections were unwrapped into row vectors and their dimensions were reduced by principal component analysis (PCA) as the input for the decoder. Finally, the behavioral outcomes of single trials were decoded using leave-one-out combined with the *k*-nearest neighbor method. The proposed method is suitable for analyzing the characteristics of LFP functional networks and for decoding behavior in single trials.

## 2. Materials and Methods

### 2.1. Subjects

Six adult pigeons* (Columba livia)* of unknown sex (450~550 g) were used in this study. All of the experiments were conducted in accordance with the Animals Act, 2006 (China), for the care and use of laboratory animals, and approved by the Life Science Ethical Review Committee of Zhengzhou University. Drug usage in the experiments complied with the Chinese Pharmacopoeia (2010 edition), approved by the Chinese Pharmacopoeia Commission. Details of behavior apparatus and experimental protocols of the pigeons are described elsewhere [27]; here we only do a brief introduction.

### 2.2. Behavioral Apparatus and Protocol

**Figure 1:**
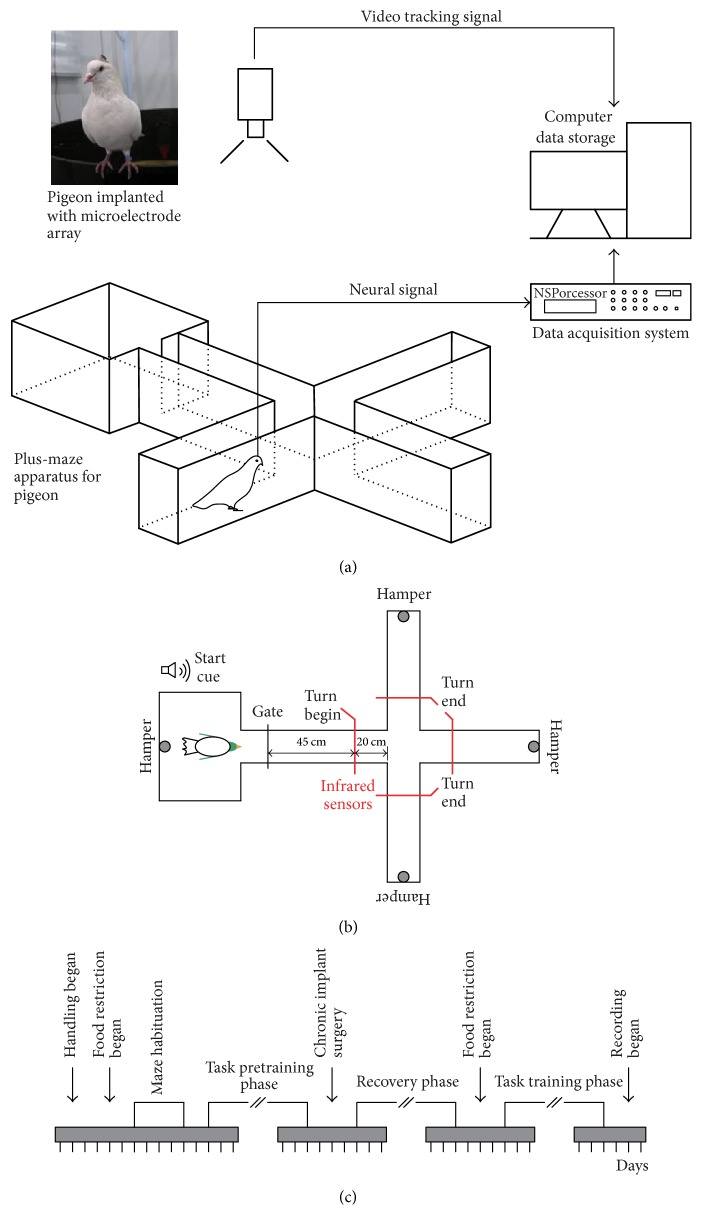
Behavioral task and experiment procedures. (a) Diagram showing the experimental setup, which comprised a pigeon implanted with a microelectrode array in a plus maze, the Cerebus data acquisition system, and the NeuroMotive™ video recording/tracking system. (b) Diagram showing the goal-directed task, where the red lines denote the positions of infrared detectors. (c) Timeline of the various phases of the experiment.

Figure 1 shows behavioral task and experiment procedures. Pigeons performed a goal-directed decision-making task in a plus maze, which included a waiting area, a choice arm, and three reward arms (Figure 1(a)). There was an automatic door between the waiting area and choice arm as well as four automatic food hampers at the waiting area and the ends of three reward arms (Figure 1(b)). Besides, four infrared sensors were located in the choice arm and in the three reward arms close to the intersection. The infrared sensor in the choice arm was defined as the turning begin sensor (TBS) and the infrared sensor in the reward arm was defined as the turning end sensor (TES). In the task, pigeons were trained to turn left, move forward, or turn right, which were random in each trial. But the times of each direction were approximately equal in a session. Pigeons ran from the waiting area to the reward arms and received a food reward, indicating the completion of a trial. The pigeons had to go back to the waiting area to start the next trial after the reward was consumed. If the trial was performed correctly, the pigeon received an additional reward in the waiting area. If a pigeon performed reliably in the trial, where the required correct attempt rate was more than 90% on two consecutive days, it was considered ready for electrode implantation. The experimental procedures are shown in Figure 1(c).

### 2.3. Surgery and Recordings

All surgical procedures were performed, while the animals were under general anesthesia. The head was placed in a stereotaxic holder, which was customized for pigeons, with the anterior fixation point (i.e., beak bar position) 45° below the horizontal axis of the instrument. Data were collected using 8- or 16-channel nickel-chromium microwire arrays (Hong Kong Plexon Inc., Hong Kong, China), which were chronically implanted in the left NCL area (a 5.5 mm; L 7.5 mm; D 2.5~4.5 mm), according to the atlas provided by Karten and Hodos [28]. Following 5~7 days of recovery, neural signals were recorded from the avian NCL with a Cerebus™ recording system (Blackrock Microsystem Inc., Salt Lake City, USA). The signals were amplified (4000x), filtered (0~250 Hz), and sampled continuously at 2 kHz, before saving the files as LFPs. The LFPs were downsampled to 1 kHz after removing the baseline wander. To minimize potential contamination by power line noise, the LFPs were filtered using an adaptive common average reference method to remove the spatially correlated artifacts [29].

### 2.4. Data Analysis and Statistics

**Figure 2:**
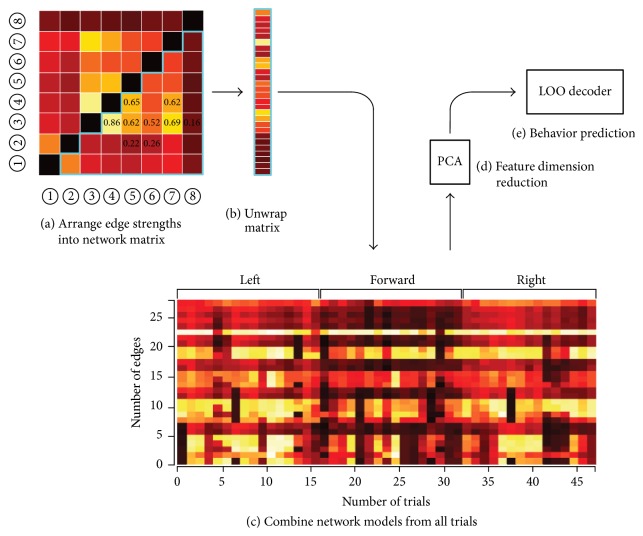
Flowchart illustrating neural functional network decoding. First, the feature bands in the LFP were extracted by wavelet filtering according to the time-frequency spectral analysis results. The functional connectivities among LFPs were then calculated and the connection strengths were arranged in a network matrix. Second, the connection strength values from the matrix were reshaped into row vectors, before they were combined with the equivalent connection strengths in all the other trials. Third, the dimension of the trials × edges matrix, which represented all the connection strengths from all the trials, was reduced using the PCA algorithm. Finally, a leave-one-out (LOO) decoder used the matrix with reduced dimensions and the predicted measures to train a set of weights, before the trained weights were combined with the connection strengths matrix to generate a single prediction (one value per trial, i.e., the movement direction of pigeons) of the behavioral measure.

We recorded LFP signals from NCL in pigeons, while they performed the goal-directed decision-making task in a plus maze. In this study, we only analyzed the data with a completion time < 60 s in a trial and correct trial data. The number of electrodes, electrode types, number of channels, and number of trials per pigeon are shown in Table 1.  Figure 2 shows flowchart of neural functional network decoding. The decoding algorithm includes determination of dominant frequency band, functional network construction, feature extraction, and decoding, specific as follows. All data analyses were performed using MATLAB 7.14 (r2012a) by MathWorks (The MathWorks Inc., Natick, USA).

#### 2.4.1. Frequency Spectral Analysis

Spectral analysis was used to assess the dominant frequency bands in the LFPs. To illustrate the temporal modulation of energy in different frequency bands, the LFP time-frequency spectrum was calculated using Complex Morlet's wavelet with 1 Hz resolution. And the LFP power spectrum was performed using the multitaper method with the time-bandwidth parameter nw = 2. The dominant frequency band was extracted by a wavelet filter due to its advantage of wavelet transform in nonstationary signal analysis. A quadratic B-spline mother wavelet was used as the wavelet filter because it has compact support, while it is smooth, symmetric, and, most importantly, it can provide optimal time-frequency resolution [30, 31].

#### 2.4.2. Network Construction

Using the characteristic frequency band, the functional connectivity of LFPs was constructed using the SL method. Although there is many methods for measure of functional connectivity, such as granger, cross-correlation, multivariate regression model, and S-transform, the SL is arguably the most popular index for estimating functional connectivity in neurophysiological data [26, 32]. It provides a nonlinear estimate of the dynamical interdependencies and relies on the detection of simultaneously occurring patterns, which can be complex and very different among signals.

If we suppose that the microelectrode array records *M* channels for LFP signals in a trial, *x*_1_(*t*), *x*_2_(*t*),…, *x*_*M*_(*t*), *t* = 1,2,…, *N*, then the corresponding *d*-dimensional delayed vectors at time *n* are defined as

(1) $$\begin{array}{c} x_{1,n}=\left(\;x_{1}\left(\;n\;\right),x_{1}\left(\;n-\tau \;\right),\ldots ,x_{1}\left(\;n-\left(\;d-1\;\right)\tau \;\right)\;\right)\\ x_{2,n}=\left(\;x_{2}\left(\;n\;\right),x_{2}\left(\;n-\tau \;\right),\ldots ,x_{2}\left(\;n-\left(\;d-1\;\right)\tau \;\right)\;\right)\\ \vdots \\ x_{M,n}=\left(\;x_{M}\left(\;n\;\right),x_{M}\left(\;n-\tau \;\right),\ldots ,x_{M}\left(\;n-\left(\;d-1\;\right)\tau \;\right)\;\right), \end{array}$$

where *τ* is the delay time. The probability that two embedded vectors from an LFP *x*_*m*_(*t*) (*m* = 1,2,…, *M*) are closer to each other than a given distance *ε* at time *n* is given by

(2) $$\begin{array}{c} P_{m,n}^{\varepsilon }=\frac{1}{2\left(w_{2}-w_{1}\right)}\overset{N}{\underset{j=1,w_{1}<\left|n-j\right|<w_{2}}{\sum }}\Theta \left(\;\varepsilon -\left|\;x_{m,n}-x_{m,j}\;\right|\;\right), \end{array}$$

where |·| is the Euclidean distance operator; *w*_1_ is the Theiler window, which is used to avoid an autocorrelation effect on the calculations; and *w*_2_ is a window that sharpens the time resolution of the synchronization measure. The values of *w*_1_ and *w*_2_ should satisfy *w*_1_ ≪ *w*_2_ ≪ *N* [33], and *w*_1_ should be at least in the order of the autocorrelation time. Θ(*x*) is the Heaviside step function as follows:

(3) $$\begin{array}{c} \Theta \left(\;x\;\right)=\left\{\begin{array}{cc} 0, & x\leq 0,\\ 1, & x>0. \end{array}\right. \end{array}$$

For each of the *M* LFP signals and each time *n*, the critical distance *ε*_*m*,*n*_ is determined for *P*_*m*,*n*_^*ε*_*m*,*n*_^: that is, *P*_*m*,*n*_^*ε*_*m*,*n*_^ = *P*_ref_ ≪ 1, where *P*_ref_ denotes the percentage of reconstructed state vectors in *x*_*m*_(*t*) that are sufficiently close to *x*_*m*,*n*_ to be regarded as dynamically equivalent to them. For each discrete time pair (*n*, *j*), the number of channels, *H*_*n*,*j*_, where the embedded vectors *x*_*m*,*n*_ and *x*_*m*,*j*_ are closer together than this critical distance *ε*_*m*,*n*_ within the time window *w*_1_ < |*n* − *j*| < *w*_2_, is

(4) $$\begin{array}{c} H_{n,j}=\overset{M}{\underset{m=1}{\sum }}\Theta \left(\;\varepsilon_{m,n}-\left|\;x_{m,n}-x_{m,j}\;\right|\;\right), \end{array}$$

where 0 < *H*_*n*,*j*_ < *M* denotes how many of the embedded signals “resemble” each other. Then, for each channel *m* and discrete time pair (*n*, *j*), the SL, *S*_*m*,*n*,*j*_, is defined as

(5) $$\begin{array}{c} S_{m,n,j}=\left\{\begin{array}{cc} \frac{H_{n,j}-1}{M-1}, & if\;\;\left|x_{m,n}-x_{m,j}\right|<\varepsilon_{m,n},\\ 0, & if\;\;\left|x_{m,n}-x_{m,j}\right|\geq \varepsilon_{m,n}. \end{array}\right. \end{array}$$

By averaging over all *j*, we finally obtain the SL, SL_*m*,*n*_:

(6) $$\begin{array}{c} SL_{m,n}=\frac{1}{2\left(w_{2}-w_{1}\right)}\overset{N}{\underset{j=1,w_{1}<\left|n-j\right|<w_{2}}{\sum }}S_{m,n,j}, \end{array}$$

where SL_*m*,*n*_ describes how strongly channel *x*_*m*_(*t*) at time *n* is synchronized with all the other *M* − 1 channels. An SL value for the *m*-th channel, SL_*m*_, is considered by averaging SL_*m*,*n*_ for all times *n*. For *M* channels in LFP signals, *P*_ref_ ≤ SL ≤ 1 [32], SL = *P*_ref_ denotes that all *M* LFPs are uncorrelated, and SL = 1 denotes the maximal synchronization of all *M* LFPs.

Calculation the SL of all channels, the functional connectivity matrix is obtained by

(7) $$\begin{array}{c} G=\left[\begin{array}{cccc} SL_{1,1} & SL_{1,2} & \cdots & SL_{1,M}\\ SL_{2,1} & SL_{2,2} & \cdots & SL_{2,M}\\ \vdots & \vdots & \ddots & \vdots \\ SL_{M,1} & SL_{M,2} & \cdots & SL_{M,M} \end{array}\right], \end{array}$$

where SL_*i*,*j*_ = SL_*j*,*i*_ and SL_*i*,*i*_ = 0. In this study, the SL indexes for LFP signals are calculated by the HERMES toolbox [32].

#### 2.4.3. Feature Extraction

To extract the network features of the LFP functional network, given that the functional connectivity matrix *G* is symmetric, only the lower triangle of the matrix was considered. Then, the connection strength values, obtained from the lower-triangular matrix, are reshaped into a row vector: that is,

(8) F=SL1,2,…,SL1,M︸M−1,SL2,3,…,SL2,M︸M−2,…,SLM−1,M︸1,

where the length of the vector *F* is *M*(*M* − 1)/2.

For *T* trials, a new functional connectivity matrix *G*′ is obtained by combining with the equivalent connection strengths from all trials:

(9) $$\begin{array}{c} G^{\prime }={\left[\;F_{1}^{T},F_{2}^{T},\ldots ,F_{T}^{T}\;\right]}^{T}, \end{array}$$

where *F*_*i*_ denotes the functional connectivity vector in the *i*-th trial, *i* = 1,2,…, *T*. The dimension of *G*′ is *T* × *M*(*M* − 1)/2, that is, trials × edges. The dimension of *G*′ is then reduced using the PCA algorithm. The first few principal components (PCs) where their sum accounts for more than 90% of the total variance in energy in the original data [34] are selected to generate the new functional connectivity matrix (*G*′′), which is defined as a network feature. Here, the PCA algorithm was performed using the function* princomp* in MATLAB.

#### 2.4.4. Decoding Algorithm

For the matrix *G*′′, leave-one-output decoding was performed with the *k*-nearest neighbor method [35]. Specifically, for each trial, the event type was decoded based on the distribution of all the remaining trials. To illustrate the neural decoding of LFP, *G*′′ can be rewritten as follows:

(10) $$\begin{array}{c} G^{\prime \prime }={\left[\;{SL}_{1}^{\prime },{SL}_{2}^{\prime },\ldots ,{SL}_{T}^{\prime }\;\right]}^{T}, \end{array}$$

where SL_*i*_′ = [SL_*i*,1_′, SL_*i*,2_′,…,SL_*i*,*C*_′]^*T*^ denotes the *i*-th trial and *C* is the number of the extracted PCs. We also assumed that *C* PCs could encode common behavioral events in each trial, that is, turning left, moving forward, or turning right. Thus, for all *T* trials, the behavior events can be expressed as

(11) $$\begin{array}{c} E={\left[\;E_{1},E_{2},\ldots ,E_{T}\;\right]}^{T}, \end{array}$$

where *E*_*i*_ ∈ {*L*, *F*, *R*}, *L* denotes turning left, *F* is moving forward, and *R* is turning right.

In the decoding algorithm, we had *T* pairs (SL_*i*_′, *E*_*i*_). If a trial was selected as the test dataset, then (*T* − 1) other trials were in the training dataset, and the training dataset was labeled as the class. Next, the class of the test dataset was decoded using the *k*-nearest neighbor (*k*NN) method. We repeat this step until the classes of all *T* trials are predicted. Finally, the *T* predicted labels and their real labels are compared, and the accuracy is calculated as follows: Accuracy = (*T*_right_/*T*) × 100%, where *T*_right_ is the number of correct predictions.

The *k*NN algorithm is a nonparametric classification method that classifies points based on actual examples in the training set [35]. Each point in the test set is assigned the same class as the majority vote of its *k*NN in the training set. Therefore, there is little or no prior knowledge about the distribution of data points. In this study, the dataset was split up into two partitions, where one partition was used as a training set and the other partition was used as a test set. The *k* value was always less than the square root of the size of training samples [36]. The *k*NN algorithm was performed using the function* knnclassify* in MATLAB.

#### 2.4.5. Network Properties

To measure the properties of the functional networks during the goal-directed task, the global efficiency (*E*_glob_) and clustering coefficient (*C*_clu_) were calculated, which described the global properties of the network. Before calculating the network properties, traditional methods usually involve converting the network into a binary network, which is formed by a threshold. The value in the matrix larger than the threshold is set to be 1, while less than the threshold is 0. The threshold is difficult to choose [37]. In order to improve analysis, we directly analyzed the weighted functional connection matrix.

*E*_glob_ is a measure of the capacity of nodes to propagate information in parallel across a network, which is defined mathematically as [38]

(12) $$\begin{array}{c} E_{glob}=\frac{1}{N\left(N-1\right)}\sum_{i,j\in N_{s},i\neq j}d_{i,j}^{-1}, \end{array}$$

where *N* is the number of nodes, *d*_*i*,*j*_ is the shortest path length between node *i* and *j*, and 0 ≤ *E*_glob_ ≤ 1. A network with a high *E*_glob_ suggests the existence of a strong association between nodes. Thus, information is communicated with a high level of efficiency throughout the network.

*C*_clu_ is a measure of the degree to which the nodes in a network tend to cluster together, which is computed by [38]

(13) $$\begin{array}{c} C_{clu}=\frac{1}{N}\sum_{i}\frac{2t_{i}}{k_{i}\left(k_{i}-1\right)}, \end{array}$$

where *N* is the number of nodes, *k*_*i*_ is the degree of node *i*, and *t*_*i*_ = 0.5 × ∑_*j*,*h*∈*N*_SL_*i*,*j*_SL_*i*,*h*_SL_*j*,*h*_, where *i* ≠ *j* ≠ *h*, 0 ≤ *C*_clu_ ≤ 1. A larger value for *C*_clu_ denotes the more close the network connected and the higher the efficiency of information transfer between the nodes.

#### 2.4.6. Statistical Analysis

For the network measures, the indexes used in the text and figures are expressed as the mean ± standard deviation (s.d.). Statistical differences were evaluated with a Wilcoxon rank-sum test, where the significance level was set to 5%. *p* values were considered to indicate significant differences at *p* < 0.05. Statistical analyses were conducted using the MATLAB Statistics and Machine Learning Toolbox.

## 3. Results

### 3.1. Spectral Properties of LFPs during the Goal-Directed Task

**Figure 3:**
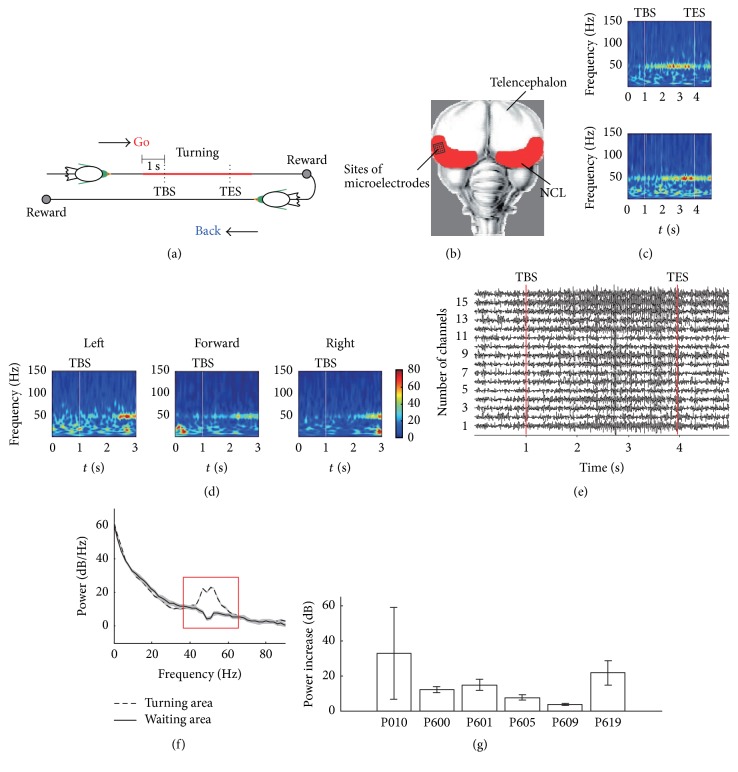
LFP spectral properties during the goal-directed task. (a) Illustration of the event window, which is marked by a colored line. TBS, turning begin sensor; TES, turning end sensor. (b) Diagram showing the microelectrode array and implanting location. (c) Time-frequency representation of a single electrode (top) and all the electrodes averaged (down) during a single trial. (d) Averaged time-frequency representation across all the trials for turning left, moving forward, and turning right. (e) Examples of LFP waveforms during a trial. (f) Example of the power spectrum of a typical recording channel in the turning area (−3 s to TES, dashed line) and the waiting area (3 s before the gate opened, solid line) in all trials (*n* = 65). (g) Mean relative power of all trials in the gamma-band (40~60 Hz) for all six pigeons.

First, we analyzed the LFPs and the spectral properties recorded from the pigeon NCL during the goal-directed decision-making task, where the results are shown in Figure 3. Illustration of both the event window and the implanting location of microelectrode array are showed by Figures 3(a) and 3(b), respectively.  Figure 3(c) shows time-frequency spectrum of a single electrode and averaged time-frequency spectrum across all the electrodes during a trial. The signals below 8 Hz were filtered.  Figure 3(d) shows the averaged time-frequency spectrum across all the trials for turning left, moving forward, and turning right. A 3 s window was used and time 0 indicated the moment when the pigeon was at 1 s before the TBS.  Figure 3(e) shows examples of LFP waveforms (gamma-band) during a trial. A dominant high-frequency oscillation was observed around the TES, which indicates that the high-frequency oscillations in the LFP may be related to the behavior of pigeons.

The LFP power spectra were calculated for the baseline period (3 s before the gate opened) and the turning period (3 s after the TBS).  Figure 3(f) shows the power spectra of a single electrode in all trials, which indicates that a broad band increased significantly in the turning area compared with the baseline in the waiting area located in a band centered around 55 Hz. At most of the recording sites, the LFP power during turning increased by 3.5-fold in the gamma-band (40~60 Hz) of the frequency spectrum (Figure 3(g)). The increase in the power value was dependent on the pigeon (mean power increase: P010 = 33.9 dB, P600 = 12.1 dB, P601 = 15.0 dB, P605 = 7.6 dB, P609 = 3.5 dB, and P619 = 22.4 dB), but an increase in the gamma-band power was prominent in all six pigeons.

### 3.2. Functional Connectivity Properties of LFP Networks

**Figure 4:**
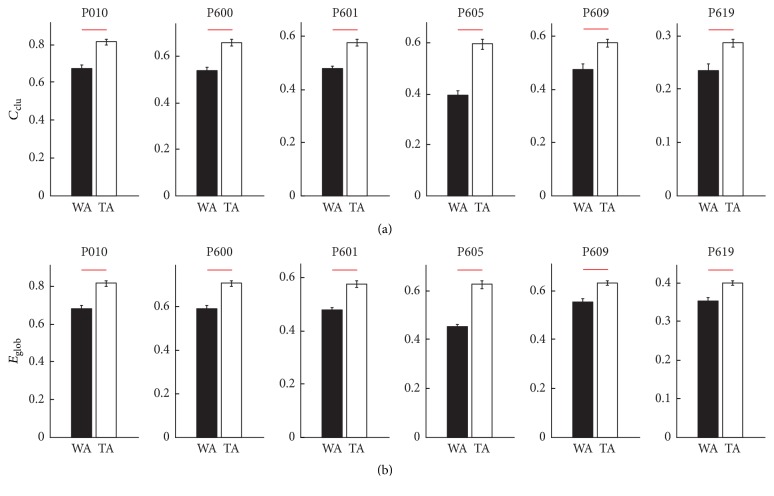
The values of *C*_clu_ and *E*_glob_ in the waiting area (WA) and the turning area (TA). (a) The values of *C*_clu_ in WA and TA. (b) The values of *E*_glob_ in WA and TA. The red lines denote *p* < 0.001 (Wilcoxon rank-sum test).

To measure the functional connectivity properties of LFP networks, we obtained the gamma-band of the LFP using a wavelet filter. The mother wavelet is bior 2.2 and the decomposition scale was equal to 4. Therefore, the frequency range of the extracted gamma-band was 31~62 Hz. The LFP network was then constructed using the gamma-band of the LFP. The functional connectivity features of the LFP network were measured by calculating the global efficiency and the clustering coefficient. We found that both *C*_clu_ and *E*_glob_ increased significantly in all six pigeons (Figure 4; Wilcoxon rank-sum test, *p* < 0.001). The results were similar as the power increased. Therefore, we propose that the LFP network may be related to the goal-directed behavior of pigeons.

**Figure 5:**
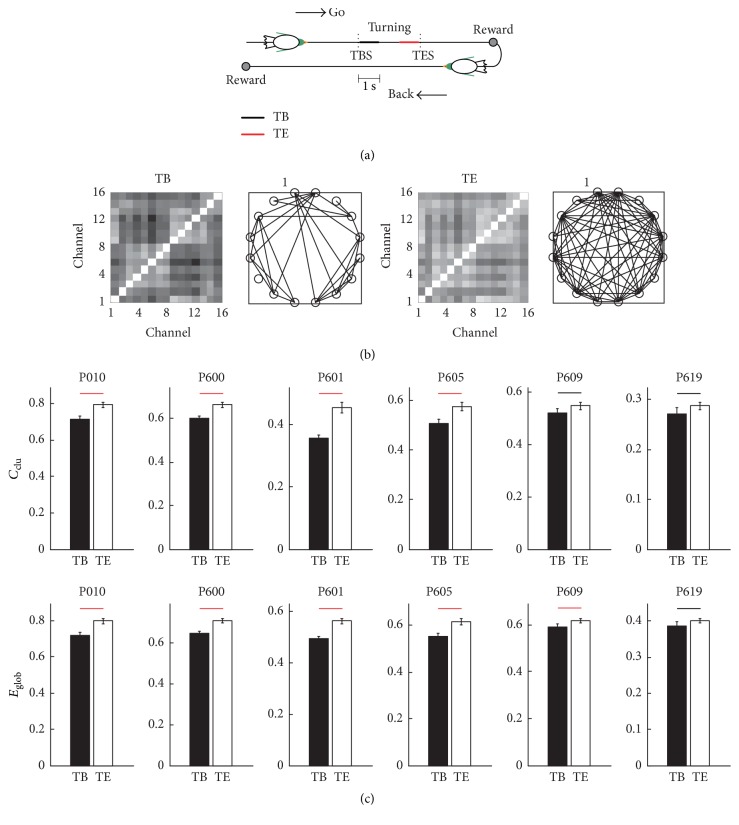
LFP functional connectivity properties. (a) Illustration of the different event windows, which are marked by a colored line. TB, 1 s after TBS; TE, 1 s before the TES. (b) Example of the functional connectivity matrixes obtained from the LFP network in TB and TE. The threshold was set to 0.78, which is the maximum value possible needed to ensure network connectivity. (c) *C*_clu_ and *E*_glob_ in TB and TE. The red lines denote *p* < 0.001 and the black lines denote *p* < 0.05 (Wilcoxon rank-sum test).

To determine the decoding window for pigeon behavior outcomes, we calculated *C*_clu_ and *E*_glob_ for the LFP network before and after turning, that is, 1 s after TBS (TB) and 1 s before TES (TE), and the results are shown in Figure 5. Illustration of the different event windows was showed by Figure 5(a). Compared with TB, the connectivity of TE was stronger (Figure 5(b)). And *C*_clu_ and *E*_glob_ were significantly higher in TE than TB (Wilcoxon rank-sum test, *p* < 0.05, Figure 5(c)). These results indicate that the functional connectivity was related more strongly to the goal-directed behavior of pigeons in TE than TB. Therefore, TE was used to decode the pigeon behavior outcome in a single trial.

### 3.3. Decoding Pigeon Behavior Outcomes

According to the results described previously, changes in the neuronal functional network during turning were related to the behavioral choices of pigeons. However, it was not clear whether these neuronal functional networks could be employed to decode the behavioral choices in a single trial. Thus, we decoded the pigeon behavior outcome in a single trial using the network features in the TE and we compared the results with those obtained using the power features, which are employed widely for behavior prediction. The power features were obtained as follows. The power of the gamma-band from all channels was first calculated in the TE and for all trials; the power dimensions were reduced using the PCA algorithm. The power with decreased dimensions was defined as the power feature.

**Figure 6:**
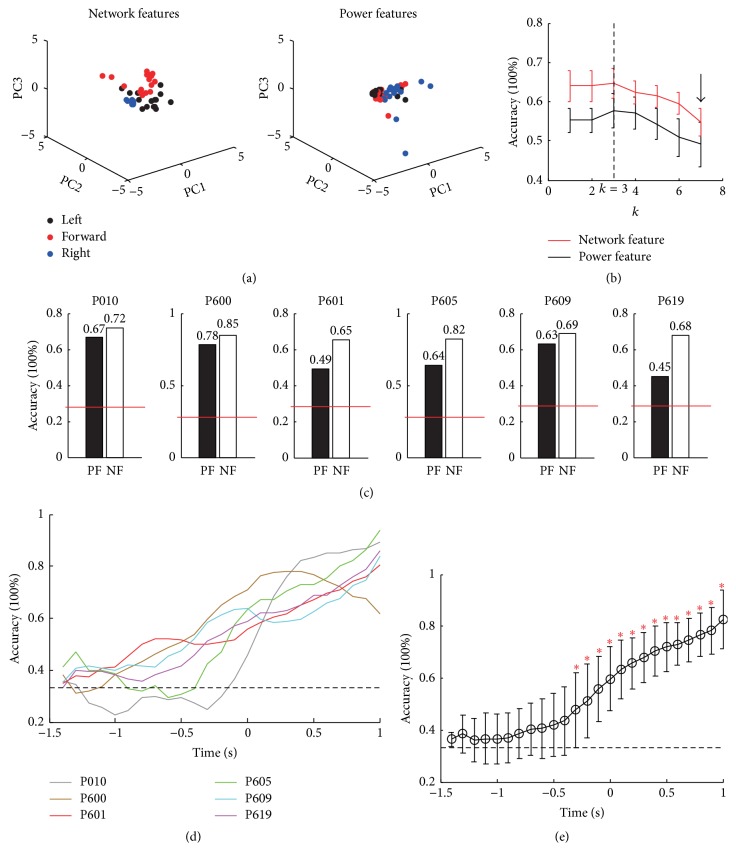
Predicted trial outcomes in the goal-directed decision-making task. (a) Feature spaces of both network features and power features. To reduce the dimensionality of features, only the first three principal components (PC1, PC2, and PC3) are shown, and the data were normalized using the *z*-score method. (b) Dependence of the mean decoding accuracy (*n* = 6) on the nearest neighbor *k*. (c) Comparison of the decoding accuracy between the power features and the network features when *k* = 3. The red dotted lines denote the channel level (0.33), and the value above the bar is the accuracy. (d) Time profile of the decoding accuracy using a (half-overlapping) moving window of 100 ms from the six pigeons. Values were smoothed using a 3-point moving average. The dotted line denotes the channel level. (e) Time profile of the decoding accuracy averaged across all pigeons (*n* = 6). The red asterisks denote *p* < 0.05 according to the Wilcoxon rank-sum test.

Figure 6 shows the decoding results obtained using network features and power features. As shown in Figure 6(a), the separability of the network features in the three directions was better than that of the power features. The accuracy of network features was significantly higher than that of the power features with different values of *k* (Figure 6(b); 1 ≤ *k* ≤ 7) except *k* = 7, which is denoted with arrow. Because the mean size of training samples is about 50 (see Table 1; 301/6), the maximum *k* value was set to 7. The largest accuracy was obtained with *k* = 3.  Figure 6(c) shows the decoding accuracy using power feature and network features when *k* = 3 for the six pigeons (power features = 61 ± 12%; network features = 74 ± 8%; Wilcoxon rank-sum test, *p* < 0.05). The results have shown that the LFP functional connections from the NCL could be used to decode the pigeon behavior outcomes of single trials, and the accuracy of the network features was better than that of the power features.

Next, we studied the time profile of the decoding performance based on the network features. Decoding was performed using a sliding window with a width of 1 s and a step size of 100 ms [10]. Time 0 indicated the moment when the pigeon was at the TES point. The accuracy peaked at the TES, except in P600, and the maximum of all pigeons was above the 80% (Figure 6(d)). The decoding accuracy differs significantly from the channel level (0.33) at the beginning, 0.7 s before the TES (Figure 6(e); Wilcoxon rank-sum test, *p* < 0.05), thereby confirming that the reward may also play a crucial role in the goal-directed behavior of pigeons.

## 4. Discussion

In the present study, we explored the functional connectivity properties of LFPs recorded from the pigeon NCL during a goal-directed decision-making task. We developed a network-based feature extraction method based on the SL index to decode the behavior of animals. We found that the power of the gamma-band increased significantly during turning by pigeons and the LFP functional network based on the gamma-band correlated with the goal-directed behavior of the pigeons. Moreover, the functional connectivity strength values among LFPs could decode the behavioral choices of pigeons in an effective manner. The accuracy of network features was significantly higher than that of power features as well as the chance level. The results showed that the proposed method is effective for decoding the task-related behavior of animals based on the functional networks obtained from multielectrode recordings.

In this study, the experimental results were controlled by six parameters: embedding dimension (*d*), embedding delay (*τ*), Theiler window (*w*_1_), *w*_2_ and *P*_ref_ for the SL index, and the nearest neighbor *k* in the *k*NN method. The first five parameters directly affected the structures of the neuronal functional networks. In this study, the SL index was set according to previously described parameter settings in the HERMES toolbox [32], so here we do not provide full details of these parameters. The nearest neighbor, *k*, controlled the decoding results, where we found that a smaller value for *k* obtained better decoding performance in the experiment: that is, the performance was generally better when *k* ≤ 3. However, comparing with the power features, the network features have better performance for all *k* values.

Decoding animal behavior based on the activity patters of neurons is a key issue in neuroscience. Previous decoding approaches studied how relevant information about the stimuli or events is represented by the neuronal population activity [39]: for example, predicting the position of a rat in its environment from recordings in hippocampus [40], arm movements from motor cortex in human [41], and image presentations from spiking activity in human medial temporal lobe [39]. Here, we demonstrated the feasibility of decoding behavior outcomes of pigeon using LFP functional connections in the NCL. Functional connective network, which contains information about the correlations between neurons or channels, may be an efficient method to decode the behavior of the animals [37]. By applying the SL method to determine the correlation between all pairwise of LFP signals, we found that the functional connectivity strength values can distinguish the behavior outcomes of pigeons, and the accuracy was significantly higher than that of power features.

Similar to the proposed method, Rosenberg et al. [42] used the strength values of brain connections based on fMRI data to predict a subject's attention capacity. By contrast, we used the functional connectivity strengths based on the LFP data obtained from the NCL to decode the behavioral outcomes of pigeon in this study. The dimensions of the connectivity strengths were also reduced by PCA, thereby improving the computational speed and avoiding the curse of dimensionality. Lu et al. [11] used the spike data from the PFC and hippocampal CA1 regions to build whole-recorded neuronal functional networks and then divided these networks into local neuronal circuit groups based on the maximization of modularity Q to predict rat behavior. Our study, which used the functional connectivity strength values among the LFP data to decode the pigeon behavior, expanded upon the work, and the results add to the growing body of evidence that neuronal functional network which exists in the brain of animals can decode the behavior of animals effectively.

Goal-directed decision-making is a complex behavior, requiring the subject to perceive its environment, learn about the significant of the environment, and then select where to go next [43]. Although rodents are classic model animals for the study of goal-directed behavior [15, 16], the wealth of available behavioral and neuroanatomical data renders the pigeons a highly suitable model system. Moreover, we have demonstrated experimentally that the NCL plays an important role during the goal-directed behavior of pigeons using the spike signals [27]. The results of this study are in line with the conclusion. The functional connections from LFP's gamma-band signals in the NCL decoded effectively the pigeon behavioral outcomes during the goal-directed task. But whether the functional connections from the spike signals could also decode the behavioral outcomes needs further study.

In addition, it should be noted that the number of recorded channels was limited in our experiments. The greater the number of channels is recorded, the clearer the relationships between the neural functional networks and behavior could be revealed [11]. But for the SL method, two important limitations for the practical use are its computational and memory costs of current implementations. As the number of channels increases, both the computational and the memory costs increase sharply. Moreover, the decoding approach used was relatively simple; the complex classification methods may get better results. And the decoding window might also not be optimal; we found that the accuracy of the network features in some pigeons, such as P601, P605, P609, and P619, still increased after the birds passed the TES. However, the method proposed in this study still provides meaningful information that may be suitable for future analysis of large-scale neuronal networks. And the obtained results supported that the functional connections among LFPs recoded from the NCL can decode pigeon behavior outcomes.

In summary, we developed a network-based method for decoding the behavior outcomes of pigeons. In the proposed method, the LFP functional connectivity strength values with reduced dimensions are set as the input for the decoder, which avoids setting thresholds and the curse of dimensionality. Moreover, the accuracy of the network features was significantly higher than that of the power features and the chance level. The results have shown that the NCL neurons contain the information about the goal-directed behavior of pigeons, and the functional connections among LFPs could decode pigeon behavior outcomes. The proposed method provides a powerful tool for decoding animal behavior outcomes using functional networks.

## Figures and Tables

**Table 1:** Data descriptions for the six pigeons.

Pigeon ID	Number of electrodes	Electrode type	Number of channels	Number of trials
P010	16	4 × 4	16	30
P600	8	2 × 4	8	65
P601	16	4 × 4	16	51
P605	16	4 × 4	16	44
P609	16	4 × 4	16	38
P619	16	4 × 4	16	74
